# Amplification and next generation sequencing of near full-length human enteroviruses for identification and characterisation from clinical samples

**DOI:** 10.1038/s41598-018-30322-y

**Published:** 2018-08-08

**Authors:** Sonia R. Isaacs, Ki Wook Kim, Junipearl X. Cheng, Rowena A. Bull, Sacha Stelzer-Braid, Fabio Luciani, William D. Rawlinson, Maria E. Craig

**Affiliations:** 1 School of Women’s and Children’s Health, Faculty of Medicine, University of New South Wales, Sydney, NSW 2052 Australia; 2 Virology Research Laboratory, Prince of Wales Hospital, Sydney, NSW 2031 Australia; 3 School of Medical Sciences, Faculty of Medicine, University of New South Wales, Sydney, NSW 2052 Australia; 4 Systems Medicine, Inflammation and Infection Research Centre, School of Medical Sciences, Faculty of Medicine, University of New South Wales, Sydney, NSW 2052 Australia; 5 Serology and Virology Division (SAViD), NSW Health Pathology East, Department of Microbiology, Prince of Wales Hospital, Sydney, NSW 2031 Australia; 6 School of Biotechnology and Biomolecular Sciences, Faculty of Science, University of New South Wales, Sydney, NSW 2052 Australia; 7 Institute of Endocrinology and Diabetes, The Children’s Hospital at Westmead, Sydney, NSW 2145 Australia; 8 Discipline of Child and Adolescent Health, University of Sydney, Sydney, NSW 2006 Australia

## Abstract

More than 100 different enterovirus (EV) genotypes infect humans and contribute to substantial morbidity. However, current methods for characterisation of full-length genomes are based on Sanger sequencing of short genomic regions, which are labour-intensive and do not enable comprehensive characterisation of viral populations. Here, we describe a simple and sensitive protocol for the amplification and sequencing of near full-length genomes of human EV species using next generation sequencing. EV genomes were amplified from 89% of samples tested, with C_t_ values ranging between 15.7 and 39.3. These samples included 7 EV-A genotypes (CVA2, 5–7, 10, 16 and EV71), 19 EV-B genotypes (CVA9, CVB1-6, ECHO3, 4, 6, 7, 9, 11, 16, 18, 25, 29, 30, and EV69), 3 EV-C genotypes (CVA19 and PV2, 3) and 1 EV-D genotype (EV70). We characterised 70 EVs from 58 clinical stool samples and eight reference strains, with a minimum of 100X depth. We found evidence of co-infection in four clinical specimens, each containing two distinct EV genotypes (CVB3/ECHO7, CVB3/ECHO18 and ECHO9/30). Characterisation of the complete genome provided conclusive genotyping of EVs, which can be applied to investigate the intra-host virus evolution of EVs, and allows further identification and investigation of EV outbreaks.

## Introduction

Enteroviruses (EVs) infect respiratory and gastrointestinal mucosa and are mostly transmitted through the faecal-oral route. Infections are often asymptomatic, but can result in a myriad of mild to severe clinical manifestations including respiratory disease, myocarditis, pancreatitis, acute haemorrhagic conjunctivitis, encephalitis, hand foot and mouth disease (HFMD), aseptic meningitis and acute flaccid paralysis^1^. Recent worldwide outbreaks of HFMD have been due to Coxsackievirus A6 (CVA6), CVA16 and EVA-71; enteric cytopathic human orphan (ECHO) viruses ECHO13, ECHO18 and ECHO30 have been associated with outbreaks of viral meningitis^2–7^; EV-D68 with severe respiratory illness and paralysis^8^; EV-A71 with pulmonary oedema in children^9,10^, and both EV species A and B have been linked to the development islet autoimmunity and type 1 diabetes (T1D)^11–13^.

EVs are ubiquitous, single-stranded RNA viruses of the *Picornaviridae* family. The EV genome consists of a single open-reading frame (ORF) between 7.2 kb and 8.5 kb in length, encoding a polyprotein of approximately 2185 amino acids. During infection, cellular and host proteases cleave the polyprotein into four structural (VP1, VP2, VP3 and VP4) and seven non-structural (2A, 2B, 2C, 3A, 3B, 3C and 3D) proteins. The 3D protein encodes an RNA-dependent RNA polymerase which plays a major role in EV replication; the lack of proofreading endonuclease activity leads to error-prone replication that contributes to the high genetic variability between EV genomes^14^. Members of a species of the genus *Enterovirus* are defined by the International Committee for the Taxonomy of Viruses (ICTV) criteria as having >70% amino acid identity in the polyprotein and >60% amino acid identity in the P1 protein, limited range of host cell receptors with a limited natural host range, and common genome organization and replication processes. To date, over 300 EV genotypes have been identified by phylogenetic clustering, and are classified into 10 EV species (A to J) and three rhinovirus (RV) species (A to C) based on their VP1 capsid protein sequences. It is important to note that excluding RVs, human-affecting EVs are classified within EV species A to D^15–17^.

Genotypes within each species are highly divergent, with prototype strains within EV species A sharing 66–86% amino acid sequence identity^18^. Phylogenies constructed from the P1 and P2-P3 genome regions of A and B species have shown frequent and recurring intra- and inter-species recombination^19^. Evolutionary changes to the EV genome including recombination, indels and substitutions contribute to genetic diversity and have impact on viral replication and pathogenicity. Recombination in the 5′UTR region rescued defective mutations introduced to pathogenic circulating vaccine-derived poliovirus (cVDPV) *in vitro*, with recombination between the cVDPV and the 5′UTR of EV-A/B species resulting in reduced viral replication and virulence, whereas recombination with the 5′UTR of EV-C/D species resulted in increased viral fitness^20^. Terminal deletions in the 5′UTR of up to 36 nucleotides which affect the domain I stem-loop are associated with an increased likelihood of viral persistence, and significantly reduced viral replication and prevent cytopathicity^21^. This naturally-occurring deletion has also been observed in virus derived from human hearts affected by EV-induced myocarditis and in the murine pancreas^22^. Additionally, a single nonsynonymous mutation (K244E) in the VP1 region of EV-A71 increased virulence and neurotropism *in vivo*^23^.

An accurate and sensitive method for virus typing is essential to investigate viral epidemiology, virulence factors and disease associations. Currently, clinical diagnosis, phylogenetic analysis and genotyping of EV infection relies heavily on Sanger sequencing of the 5′UTR and the VP1 region^24^. Application of high-throughput sequencing to instead characterise the complete EV genome sequence can allow conclusive genotyping and the discrimination between multiple EVs that may be present during co-infection, and enable identification of recombination sites or antiviral resistance mutations^17^. Detailed characterisation of EV infections may also aid in the identification of EV strains or mutations that associate with adverse clinical outcomes, such as severe neurological complications or T1D. To this end, we describe a simple method for amplifying and sequencing near full-length EV genomes from human clinical specimens.

## Results

### Optimisation of Near Full-Length Genome PCR

**Figure 1:**
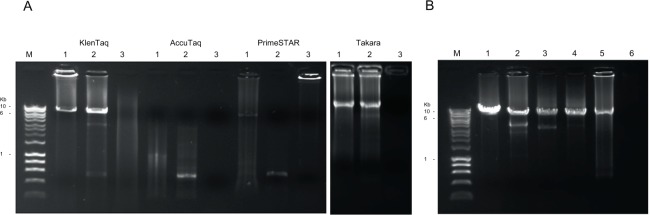
(**A**) Gel electrophoresis of near full-length genome PCR products produced from four long amplifying DNA polymerases; KlenTaq LA (discontinued), AccuTaq LA, PrimeSTAR GXL and Takara LA Taq (separate gel) per manufacturer’s instructions for samples with high GC/secondary structures. M, HyperLadder 1 kb; 1, CVB3 Nancy; 2, CVB5 Faulkner; 3, H_2_O control. (**B**) Gel electrophoresis of near full-length genome PCR products produced from Takara LA Taq DNA polymerase. M, HyperLadder 1 kb; 1–4, known EV positives from NSW Health Pathology East virology diagnostic lab; 5, CVB3 Nancy; 6, H_2_O control. Full-length gels are presented in Supplementary Fig. 1.

At the commencement of this study, KlenTaq LA (Clontech) enzyme was determined to be the optimal polymerase for near full-length EV genome PCR. However, in late 2015, production of this enzyme was discontinued. In search of an alternative enzyme, three different long amplifying polymerases, including the Takara LA Taq DNA Polymerase (Clontech), AccuTaq LA DNA Polymerase (Sigma) and PrimeSTAR GXL DNA Polymerase (Clontech) were compared against KlenTaq for their efficiency in amplifying two American Type Culture Collection (ATCC) EV prototype strains, CVB3 (Nancy; JX312064.1) and CVB5 (Faulkner; AF114383.1) (Fig. 1A). Takara LA was the only polymerase other than KlenTaq LA to successfully amplify near full-length genome products of ~8 kb, and this enzyme was used in further testing of the method using four stool specimens from the NSW Health Pathology East virology diagnostic laboratory, which were previously confirmed as EV-positive using diagnostic qPCR. As anticipated, near-full length EV amplicons were successfully amplified from all four specimens (Fig. 1B). Full-length gels are presented in Supplementary Fig. 1.

### Efficiency of Near Full-Length EV PCR

**Figure 2:**
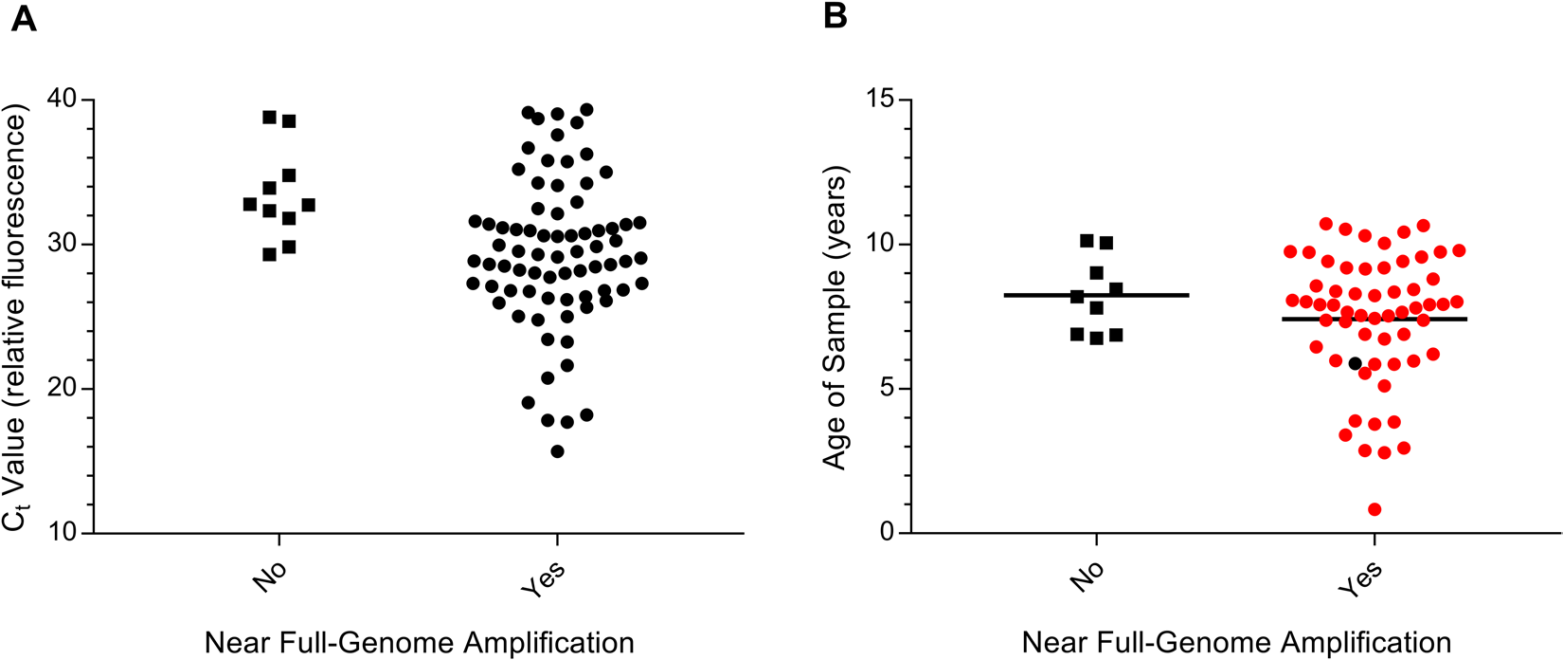
(**A**) Sensitivity of near full-length EV genome PCR tested on VIGR clinical stool samples (n = 65) and reference strains (n = 20) with EV infection detectable within 40 cycles by qPCR. PCR amplification was significantly affected by C_t_ value, with successful amplification resulting from C_t_ values ranging between 15.7 and 39.3. (**B**) Efficiency of near full-length EV genome PCR tested on VIGR clinical stool samples (n = 68) compared to sample age (years from collection to processing), with sequenced (red) and unsequenced samples (black). Mean sample age indicated. Sample age did not significantly affect outcome of amplification, with 6 samples over 10 years old producing a 7–8 kb EV amplicon using gel electrophoresis.

To evaluate the efficacy of our nested-PCR on a greater scale, we tested the method on 91 additional samples, including 68 clinical stool specimens from the Viruses in the Genetically at Risk (VIGR) cohort study^25^ and 23 purified EV reference strains. All VIGR specimens were previously confirmed as EV-positive using semi-quantitative RT-PCR, performed within 12 months of collection. Near full-length EV amplicons were successfully amplified from 87% of VIGR samples (59/68) and 96% of prototype/lab strain samples (22/23), providing an overall success rate of 89% (81/91). Amplified EVs included 7 EV-A genotypes (CVA2, 5–7, 10, 16 and EV71), 19 EV-B genotypes (CVA9, CVB1-6, ECHO3, 4, 6, 7, 9, 11, 16, 18, 25, 29, 30, and EV69) and 3 EV-C genotypes (CVA19 and PV2, 3) and 1 EV-D genotype (EV70). Additionally, EV-D68 and RV-56 were successfully amplified and sequenced from nasopharyngeal samples using this method (data not shown). Based on qPCR, the C_t_ value of successfully amplified samples ranged between 15.7 and 39.3 (Fig. 2A). The length of sample storage at −80 °C did not appear to significantly impact the success of nested PCR, with six of the successful samples stored >10 years (Fig. 2B).

### Near Full-Length EV Sequencing

**Figure 3:**
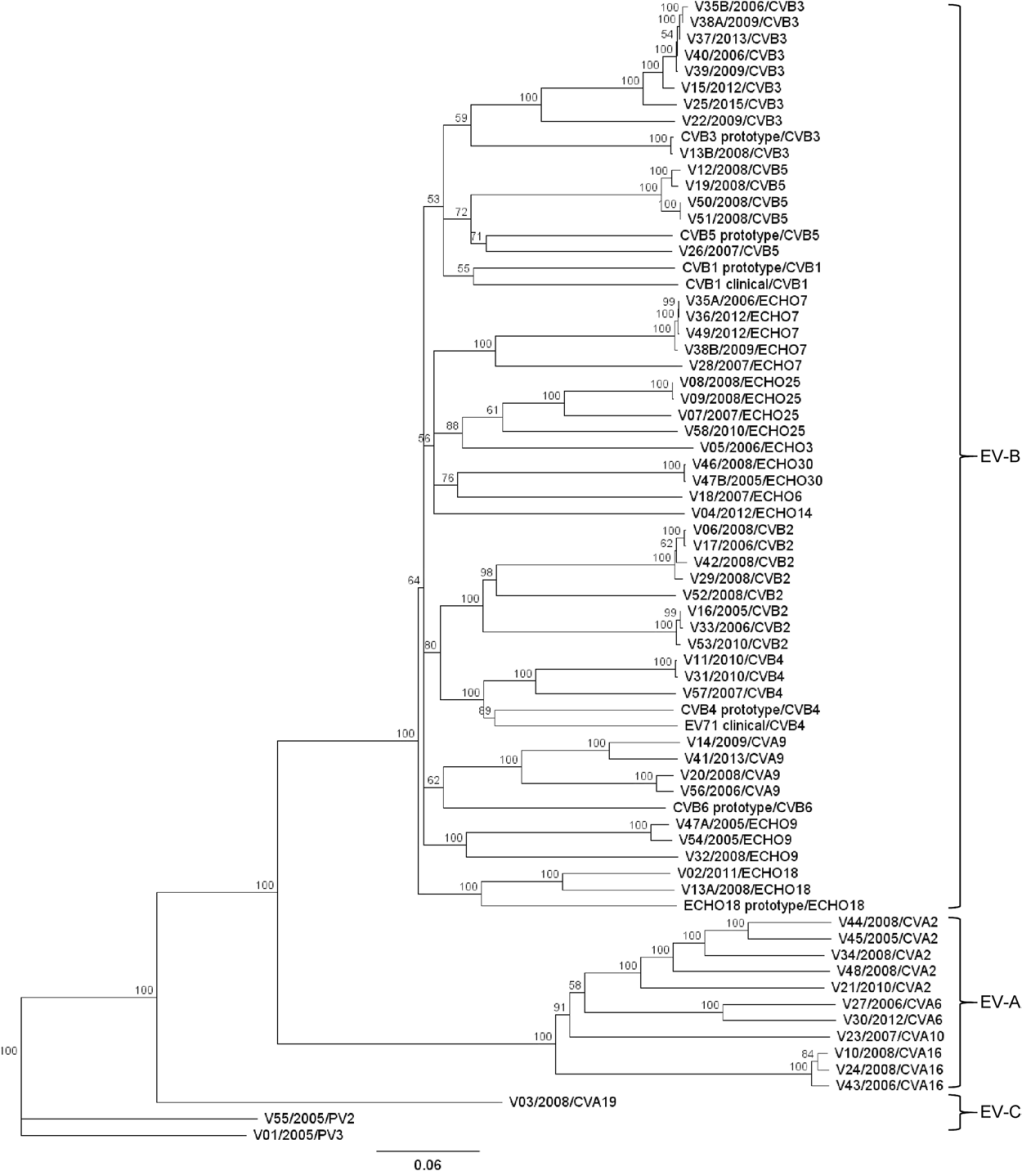
Phylogenetic analysis of the near full-length EV genomes (n = 70) from 58 clinical VIGR stool samples from VIGR children with six previously sequenced prototype strains (CVB1 clone (M16560), CVB3 ATCC Nancy (M33854), CVB4 cultured J.V.B (X05690), CVB5 ATCC Faulkner (AF114383), CVB6 cultured Schmitt (AF039205), ECHO18 cultured Metcalf (AF081331)), a CVB1 lab-cultured strain and an EV71 clinical isolate. The unrooted phylogenetic tree was constructed using the HKY genetic distance model and the Neighbour-Joining method (1000 bootstrap replicates) and displayed as rooted with PV3 outgroup selected, implemented in Geneious software package version 9 (Biomatters). Samples named in the format “sampleID/year of collection (for VIGR isolates)/top BLAST hit”. Scale bar indicates genetic distance (substitutions per nucleotide). Sample clustering into EV species A-C is also depicted. Multiple infections indicated by A and B isolates for samples V13, V35, V38 and V47.

Of the 81 near full-length EV genome fragments amplified, 66 were selected for library preparation and Illumina high-throughput sequencing. They included amplicons from 58 VIGR samples, six prototype EVs and two lab strains (CVB1 and EV71 clinical isolates). In total, 78.3 million reads were generated, with a mean of 1.2 ± 0.8 million reads per sample. Following *de novo* contig assembly, nucleotide BLAST and phylogenetic analysis confirmed that all amplicons contained sequences of EVs within species A, B or C (Fig. 3). This was determined with a minimum of 100X depth of coverage and 80% to 99.9% sequence identity to the reference genome.

Of the 31 VIGR samples where the 5′UTR or VP1 regions had been previously characterised using Sanger sequencing, 11 (35%) produced matching genotype results and 20 (65%) were identified as a different genotype when using this method. VP1 and near full-length genome sequencing produced matching genotype results in all five samples where the VP1 was sequenced, whereas 5′UTR sequencing produced matching genotypes in only 7/27 (26%) samples where the 5′UTR was sequenced. Comparison between genotypes identified using Sanger or next generation sequencing is provided in Supplementary 3.

### Multiple EV Infection

**Figure 4:**
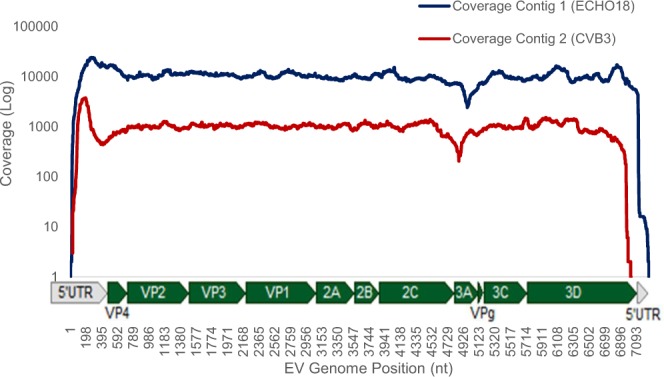
Coverage plot produced from *de novo* assembly of trimmed short reads produced from VIGR clinical stool sample 13 (V13), resulting in two Contigs over 6.9 kb, implemented in Geneious package version 9 (Biomatters). Both Contig 1 (blue) and Contig 2 (red) are shown, which were identified as ECHO18 and CVB3 respectively using BLAST. V13 displays infection with two viral genotypes at a single time point, with Contig 1 produced from 543048 reads, and Contig 2 produced from 52234 reads, which are stratified by EV genome position.

For four amplicons (V13, V35, V38 and V47), *de novo* assembly generated two major contigs >6.5 kb in length, which matched two different EV genotypes using BLAST, indicative of multiple EV infections. For example, assembly of sequence reads from V13 generated two contigs matching to ECHO18 and CVB3. ECHO18 appeared to be the dominant genotype, accounting for ~92% of the sequence reads produced from V13 (Fig. 4).

## Discussion

We have developed a simple, efficient and sensitive method to amplify near full-length EV genome products from clinical stool specimens. Coupled with NGS, this method was successfully applied to amplify EV fragments from 87% of clinical samples tested and to characterise near complete genome sequences of 22 separate EV genotypes from 58 EV-positive clinical stools that were sequenced. The upper limit for amplification using our nested-PCR was a C_t_ value of 39.3, which was the highest C_t_ value within 40 cycles of qPCR among the samples that produced near full-length EV amplicons. Nguyen *et al*. report a 71% success rate in generating near full-length EV genome sequences from 24 tested clinical rectal/throat swabs which were EV-positive with C_t_ values ranging between 20.8 and 33.3^26^.

It is important to note that the clinical stool specimens from the VIGR cohort were previously frozen and thawed at least once, and had been confirmed as EV-positive using semi-quantitative RT-PCR up to 10 years prior to this study. Therefore, the failure to amplify EV products in 13% of EV-positive stools tested could be attributed to the progressive degradation of EV RNA over multiple freeze-thaw cycles and extended period of storage, even at −80 °C. Indeed, for HCV, multiple freeze-thaw cycles can result in 16% decrease of viral titre, and 6 months of −80 °C storage resulted in ~10% loss^27^. In samples of very low viral titre, concentrating EV using ultracentrifugation prior to nucleic acid extraction may improve the success rate of the EV nested PCR.

According to current taxonomy for viruses (International Committee on Taxonomy of Viruses, 2017), the EV genus consists of four human EV species: EV-A, -B, -C and -D^15^. Despite the initial primer design against EV-B genotypes CVB1-6, alignments of primers to all human EV prototype strains indicated suitable base-pairing with a range of genotypes from each species. Our method successfully sequenced near-complete genomes of EVs belonging to species A-C. Although no EV-D genotypes were sequenced from the stool samples examined, the EV-D70 prototype was successfully amplified in this study. Therefore, it is anticipated that this method can also be effectively applied to characterise EV-D genotypes. Indeed, in our recent screening of respiratory samples collected from patients associated with neurological symptoms, we successfully amplified and sequenced one EV-D68 genotype and one RV-A56 using the same nested PCR method (unpublished data). This is important as EV-D68, formerly known as human rhinovirus 87, is an emerging pathogen that can cause severe respiratory disease such as bronchiolitis or pneumonia, especially among children^28,29^. Furthermore, during the 2014 outbreak in the United States, EV-D68 infection was associated with acute flaccid myelitis and cranial nerve dysfunction in children, implicating it as a potential public health threat^30^.

In this study, there was evidence of multiple EV infection in 4/58 clinical stool specimens examined. Coinfection of multiple EV genotypes such as CVA16 and CVB5 can result in more severe illness in some individuals^31^, but is difficult to detect using Sanger sequencing, as one virus may overshadow another due to a large difference in viral titre or preferential PCR amplification. Coinfection with genotypes within EV-A/-A, EV-B/-B, EV-A/-B, and EV-A/-C species have been previously documented in clinical stool samples, with cocirculation of different EV genotypes promoting multiple recombination events and further increasing genotype diversity^19,32^.

Recently, alternate approaches have been taken to sequence the full-length EV genome^26,33,34^. Tan *et al*. used a combination of single and dual amplicons targeting EV-D68 producing a mean of 62256 reads per sample. Nguyen *et al*. used a library of 96 non-ribosomal (rPCR) PCR amplification primers in combination with DNase pre-treatment to enhance amplification of viral reads, with EV sequences assigned to between 0.2% and 90.2% of total reads. Midgley *et al*. used a primer independent, poly(A)-capture-based method based on RNA sequencing, however this method is not EV-specific and produces non-viral reads such as those from bacteria. Our approach significantly differs as it can successfully amplify genomes of a range of EVs in a single amplicon, with a simple analysis pipeline which does not require the removal of bacterial and human reads. The high depth of coverage produced for each sequenced virus using our method allows for more powerful mutational analysis such as is required for viral SNP detection.

With rapid advances in sequencing throughput and the capacity to now multiplex up to 384 libraries per run on the Illumina platform using dual-index barcoding, the application of NGS to clinically diagnose and genotype virus infections is becoming increasingly practical and cost-effective. Compared to the standard method of genotyping EVs using Sanger sequencing which involves only a small portion of the 5′UTR or the VP1 coding region^35–37^ and, can result in ambiguous results that require repeat testing, characterising the complete genome can provide definitive, one-shot genotyping of EVs. Indeed, a clinical specimen we examined was previously genotyped as EV71 based on 5′UTR sequencing, but sequence analysis of the near full-length genome revealed that it was in fact CVB4. Furthermore, 74% of clinical stool samples produced a different genotyping result using this method compared to previous sequencing of the conserved 5′UTR region. In addition to definitive typing, our method enables comprehensive analysis of the virus phylogeny, the emergence of quasi-species and the occurrence of virus recombination. Furthermore, it has the capacity to detect multiple EV infections, and can be utilised in infection time-course experiments to investigate the intra-host evolution of EVs. Therefore, we anticipate our method to be widely useful for basic and clinical research, investigating the epidemiology and molecular behaviour of EVs.

## Material and Methods

### Study Population

Clinical stool specimens used in this study were collected from participants in the VIGR prospective cohort study, which is described elsewhere^25^. These samples were taken at clinical study visits and stored at −80 °C prior to testing. The presence of EV sequences in all samples were previously confirmed using semi-quantitative RT-PCR using primers targeting the EV 5′UTR^38^. The 5′UTR or VP1 regions of 31 samples had previously been characterised using Sanger sequencing. Mean age of children at time of sampling was 2.0 ± 1.3 years.

### Ethics Statement

Ethical approvals for the VIGR study were obtained from the Sydney Children’s Hospital Network Human Research Ethics Committee (SCHN HREC/12SCHN/225). Informed consent was obtained from all participants and/or their parents. Methods were carried out in accordance with the relevant guidelines and regulations.

### Primer Design

**Figure 5:**
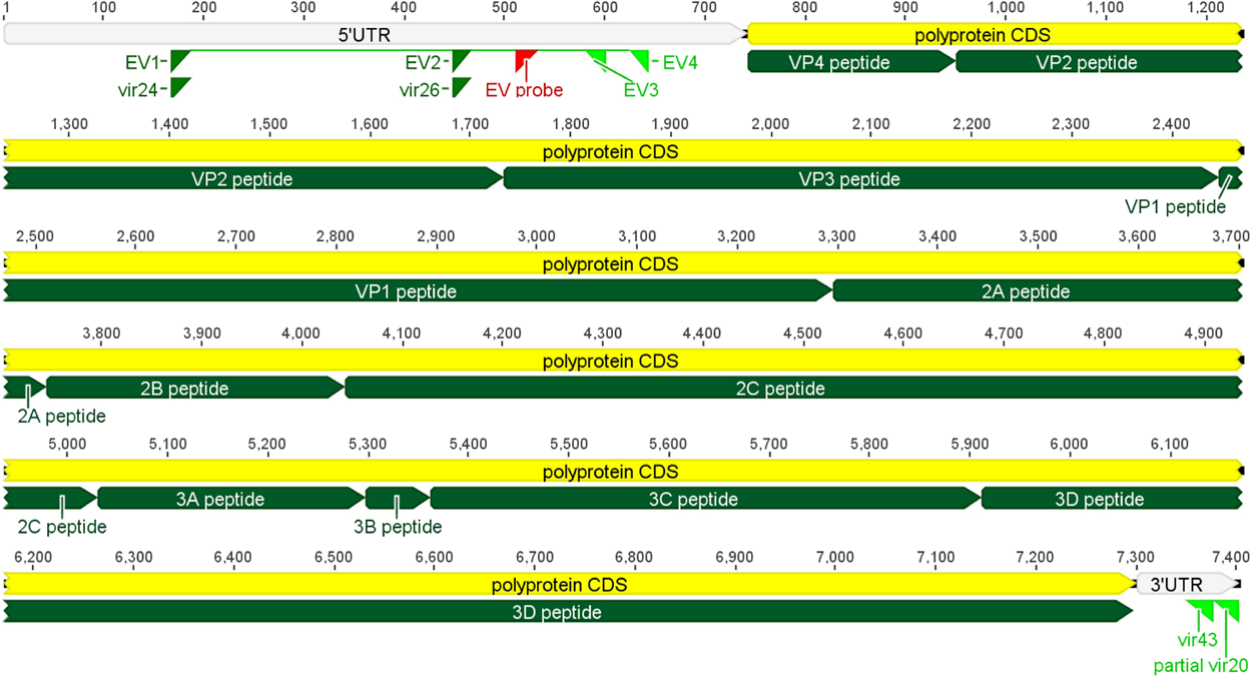
EV prototype CVB5 strain Faulkner (Accession AF114383.1) annotated with 5′UTR and near full-length genome forward and reverse primer binding sites, including EV probe and inner and outer primer sets. Viral polyprotein coding sequence (CDS) and mature peptide locations are also indicated; implemented in Geneious package version 9 (Biomatters).

For the initial primer design, full-length genome sequences representing prototype strains of CVB 1-6 genotypes were aligned using Molecular Evolutionary Genetics Analysis (MEGA) software version 7. Reverse transcriptase primer had been previously published as NotdT25^39^. Forward primers targeting the conserved 5′UTR were modified from previously published primers^38^ (Table 1). Reverse primer binding sites were selected using manual scanning of the 3′UTR for highly conserved regions (≥90% identity and >20 bp). To account for highly divergent EV strains, degenerate bases were inserted as needed to improve sequence identity. Final primer sequences were aligned against all human EV prototype strains to evaluate binding with other EV genotypes. Approximately 400 bp from the 5′ terminus was not targeted using the amplification primers. However, nested primer sets were predicted to produce a polypeptide covering the entire EV coding sequence (Fig. 5).

**Table 1:** Primers used for qPCR targeting 5′UTR and near full-length EV PCR amplification.

Region	Round	Sense	EV species target	Primer name	Sequence (5′-3′)^b^	Genome binding position^c^
NFG^a^	RT	—	All	vir21*	ATAAGAATGCGGCCGCTTTTTTTTTTTTTTTTTTTTTTTTT	—
5′UTR/NFG	qPCR/Outer	OF	All	EV1/vir24**	CAAGCACTTCTGTTTCCCCGG	167
5′UTR	qPCR	OR	All	EV4**	CACYGGATGGCCAATCCAA	645
5′UTR	qPCR	—	All	EV probeˆ	[6FAM]TGTGTCGTAACGGGTAACTCTG[BHQ1]	512
5′UTR/NFG	qPCR/Inner	IF	All	EV2/vir26**	TCCTCCGGCCCCTGAATGCG	448
5′UTR	qPCR	IR	All	EV3**	ATTGTCACCATAAGCAGCCA	602
NFG	Outer	OR	A,B,C	vir20ˆ	TTTTTTTTTTTTTTTTTTTTTTTTTCCGCACCGAATGCGGAGAATTTAC	7426
NFG	Inner	IR	A,B,C	vir43ˆ	CCCTACYRCACCGTTRTCTRGTTCGGT	7377

*Primers were adapted from Lindberg *J Vir Methods*, 1997. **Primers were adapted from Craig *J Clin Micro*, 2003. ˆPrimers unpublished.

^a^NFG; near full-length genome.

^b^For degenerate primers, R = A or G, Y = C or T.

^c^Genome binding position in reference to EV genotype CVB5 strain Faulkner, GenBank accession AF114383.1.

### Nucleic Acid Extraction

Nucleic acid was extracted and purified using the MagMAX Total Nucleic Acid Isolation Kit (Thermo Fisher Scientific) on the semi-automated KingFisher FLEX Purification System (ThermoFisher Scientific), following manufacturer’s instructions for solid samples with minor changes. Stool suspensions of 30% (w/v) were prepared in PBS (1X), and centrifuged at 13000 × g for 5 min. Cells were lysed by bead-beating using zirconium bead tubes containing Lysis/Binding Solution (235 μL) and stool suspension (175 μL). Tubes were shaken vigorously at 2400 rpm for 15 min on the Bioshake iQ, then centrifuged for 3 min. Lysate (300 μL) was transferred into new tubes and centrifuged for a further 6 min to pellet carry-over beads and cell debris. Nucleic acid extraction was performed using lysate (200 µL) combined with 100% isopropanol (100 µL) and magnetic Bead Mix (20 µL). Two washes using Wash Solution 1 (300 µL) were followed by two washes using Wash Solution 2 (450 µL). Total nucleic acid was eluted in Elution buffer (50 µL, Tris-HCl, pH 8.4) and RNAse-OUT (1 µL) was added before storing at −80 °C. RNAse-OUT was added to improve long-term RNA storage.

### Reverse Transcription

Previous RT-PCR optimisation for the amplification of near full-length Hepatitis C (HCV) genome (9.6 kb) demonstrated that the use of Superscript III and the addition of Betaine (1 M) produced optimal cDNA yield. Reduced centrifugation speeds and pipette mixing reduced RNA shearing, and storage of cDNA at −20 °C prevented sample degradation^40^. Given the similar genome size of EV (7.2-8.5 kb), we also incorporated the use of Superscript III, 1 M Betaine, reduced centrifugation speeds and storage conditions in our near full-length RT-PCR.

First-strand cDNA was synthesised from total nucleic acid using SuperScript III enzyme (Life Technologies), with a pan-genotypic reverse transcription primer (vir21, Table 1). Briefly, total nucleic acid (8 µL), 10 µM primer (1 µL) and 10 mM dNTPs (1 µL) were mixed and incubated at 65 °C for 5 min and then cooled on ice for 1 min. To the denatured nucleic acid mix, reaction mix (10 µL) was added, resulting in a final concentration of RT buffer (1X), MgCl_2_ (5 mM), Betaine (1 M), DTT (0.01 µM), RNAseOUT (40 U) and reverse transcriptase (200 U). Reverse transcription was performed using the following cycling conditions: 49 °C for 65 min, 85 °C for 5 min. RNaseH (2 U) was added immediately afterwards and the digest performed at 37 °C for 20 min. Final cDNA products were purified using the ChargeSwitch PCR Clean-Up Kit according to manufacturers’ instructions, with minor modifications. Reactions were completed using cDNA (20 µL) combined with an equal amount of Purification Buffer N5. Purified cDNA was eluted in elution buffer (30 µL) prior to storage at −20 °C.

### Enterovirus qPCR

EV positivity was initially determined using EV-specific qPCR targeting the 5′UTR, performed on the LightCycler 480 System (Roche Applied Science, Sydney). For qPCR, cDNA (4 µL) was added to freshly prepared reaction mix (16 µL) containing Sensimix II probe (12.5 µL, 2X), freshly diluted EV probe (0.25 µL, 20 µM), inner forward primer EV2 (1 µL, 10 µM), inner reverse primer EV3 (1 µL, 10 µM) and of nuclease-free H_2_O (1.25 µL) per reaction. Cycling conditions were: 95 °C for 5 min, 40 cycles at 95 °C for 5 s, 55 °C for 45 s, and 68 °C for 45 s, phase of 40 °C for 30 s. Fluorescence (465–510 nm) was measured once every cycle during extension. C_t_ values were determined using the Absolute Quantification/2^nd^ Derivative Max Method and the high sensitivity mode.

### Comparison of Polymerases for Efficient Near Full-Length EV Amplification

The CVB5 prototype strain (Faulkner, ATCC VR-185) was obtained with full-length cDNA transcribed using the above protocol. This was used as a template for full-length EV amplification PCR, to test the efficiency of three commercially available long amplifying polymerases in comparison to KlenTaq LA (Clontech), which discontinued production in late 2015^40^. The following enzymes were tested: Takara LA Taq DNA Polymerase (Clontech), AccuTaq LA DNA Polymerase (Sigma) and PrimeSTAR GXL DNA Polymerase (Clontech). These polymerases were selected due to their ability to amplify amplicons of >8 kb^41^. Reaction conditions used for tested polymerases are described in Supplementary 2.

### Amplification of Near Full-Length EV Genomes

First round PCR was performed by adding cDNA (5 µL) to a reaction mix (50 µL) containing Takara LA Buffer (1X), dNTPs (100 µM each), forward primer vir24 (0.2 µM), reverse primer vir20 (0.2 µM), nuclease-free water (29.5 µL) and Takara LA DNA polymerase (2.5 U). PCR was performed at 94 °C for 1 min, 30 cycles at 94 °C for 30 s, 60 °C for 30 s, and 72 °C for 8 min, followed by a final extension at 72 °C for 5 min. Cycling conditions for samples with high GC/secondary structures were selected, as recommended in the manufacturer’s protocol. The nested round of PCR was performed with first round product (5 µL) in a 50 µL reaction as described for the first round, except vir26 was used as the forward primer and vir43 was used as the reverse primer (see Table 1).

Samples positive for near full-length genome amplicons were identified by the presence of a band of the correct size of approximately 7.2–8.5 kb using agarose gel electrophoresis and were selected for further processing. Near full-length amplicons were purified using Ampure XP magnetic beads (Beckman Coulter) at a 1.8X (v/v) ratio according to the manufacturer’s protocol with minor modifications.

Samples were incubated with Ampure beads for 15 min at room temp, washed with freshly prepared ethanol (80%), eluted with nuclease-free water (40 µL) for 5 min and placed on the Agencourt SPRIPlate 96 Super Magnet Plate for 2 min. Sample elute was transferred to 0.2 mL PCR tubes and stored at −20 °C.

### Library Preparation for Next Generation Sequencing

Following purification, double-stranded DNA was quantified in duplicate using the NanoDrop ND 1000 spectrophotometer (NanoDrop Technologies), with the average used to dilute samples to 1 ng/µL. Sample DNA was further quantified in duplicate using the Quant-iT PicoGreen dsDNA Assay Kit according to manufacturer’s instructions, with fluorescence measured on the Victor™ X2 multimode plate reader (PerkinElmer). Calculated sample concentrations were used to dilute each sample to 0.36 ng/µL prior to library preparation. Dual-indexed Illumina sequencing libraries were prepared from purified amplicons using the Nextera XT DNA Library Preparation Kit (Illumina) according to manufacturer’s protocol with following changes. Reagent volumes were reduced including Tagment DNA Buffer (5 µL), Amplicon Tagment Mix (2.5 µL), sample DNA (2.5 µL), Neutralize Tagment Buffer (2.5 µL), Nextera PCR Master Mix (7.5 µL), index 1 (i7, 2.5 µL each) and index 2 (i5, 2.5 µL each).

Following library prep, products were purified using Ampure XP magnetic beads with 0.6X (v/v) ratio to selectively purify fragments >280 bp. Size distribution of library fragments was assessed on the LabChip® GX Touch 24, using the HT DNA High Sensitivity Assay (PerkinElmer) following the manufacturer’s protocol. Samples were pooled in equimolar amounts prior to sequencing in two runs; the first pool contained 8 prototype strains and 5 clinical isolates and the second pool contained the remaining 53 clinical isolates.

### Illumina Sequencing and EV Contig Construction

Sequencing was performed at the Ramaciotti Centre for Genomics, University of New South Wales, using a MiSeq Benchtop Sequencer generating 2 × 150 bp paired-end reads (v3 kit). De-multiplexed raw reads were imported into Geneious software package version 9 (Biomatters, https://www.geneious.com/)^42^, with forward and reverse reads paired with an expected insert size of 150 bp, and then trimmed at an error probability ratio of 0.05 to remove poor quality bases.

Contigs were generated from a random selection of 40000 trimmed reads using Geneious *de novo* assembler, with consensus sequences generated with a 0% Majority Threshold and a minimum calling coverage of 100. Large contigs over 3000 bp were extracted, and a reference-based assembly of 100% of the paired reads to each contig was performed using the Geneious Mapper with a minimum mapping quality of 30, with reads first re-trimmed to an error probability limit of 0.01. Inner forward and reverse PCR primers were aligned to the resulting consensus sequence, and the regions flanking the primers as well as low coverage ends were trimmed to improve consensus quality.

### Genotype Identification and Phylogenetic Analysis

Near full-length sample contigs were identified at a genotype level using the Basic Local Alignment Search Tool (BLAST) embedded in Geneious using the National Centre for Biotechnology Information (NCBI) Nucleotide database and the final consensus as the query sequence. The hit with the highest pairwise identity (%) and query coverage (%) was selected as the likely sample genotype. Alignments of final consensus sequences from each sample were produced using Geneious global alignment from 2 iterations, with free end gaps and a 65% similarity cost matrix selected. Unrooted phylogenetic trees were constructed from the alignments using the neighbour-joining method and the HYK genetic distance model, with 1000 bootstrap replicates incorporated. Additionally, the VP1 region of each consensus was extracted and identified using BLAST to confirm sample genotype as the highly variable VP1 region is traditionally sequenced in isolation or in combination with the VP2 for genotyping.

### Data availability

The datasets generated and analysed during the current study including near full-length EV genome sequences obtained from VIGR clinical isolates (V01-V58) are available in the GenBank nucleotide sequence repository, https://www.ncbi.nlm.nih.gov/nucleotide?cmd=search, and can be found under accession numbers MF678293 to MF678348, MF838733 to MF838737 and MF962897. For more details see Supplementary 4. Paired-reads for VIGR clinical isolates have been deposited with the NCBI Sequence Read Archive under BioProject ID PRJNA475616.

## Electronic supplementary material

Supplementary 1-4
