# Supplementary material for: Amplification and next generation sequencing of near full-length human enteroviruses for identification and characterisation from clinical samples
Source: Sci Rep. 2018 Aug 8;8:11889. doi: 10.1038/s41598-018-30322-y (PMC6082906; doi:10.1038/s41598-018-30322-y)
Supplement: Supplementary file 1 — Supplementary 1-4 [file 41598_2018_30322_MOESM1_ESM.docx]

**Amplification and next generation sequencing of near full-length human enteroviruses for identification and characterisation from clinical samples**

Sonia R. Isaacs^1,2^, Ki Wook Kim^1,2^, Junipearl X. Cheng^3^, Rowena A. Bull^3,4^, Sacha Stelzer-Braid^2,3^, Fabio Luciani^3,4^, William D. Rawlinson,^1.2.,3,5,6^ and Maria E. Craig^1,2,7,8,*^

**SUPPLEMENTARY DATA**

**Supplementary 1.A)** Gel electrophoresis of near full-length genome PCR products as presented in Figure 2 A. PrimeSTAR DNA polymerase was also tested with an altered denaturation temperature of 98°C. M, HyperLadder 1kb; 1, CVB3 Nancy; 2, CVB5 Faulkner; 3, H_2_O control.

KlenTaq AccuTaq PrimeSTAR PrimeSTAR 98°C

M 1 2 3 1 2 3 1 2 3 1 2 3


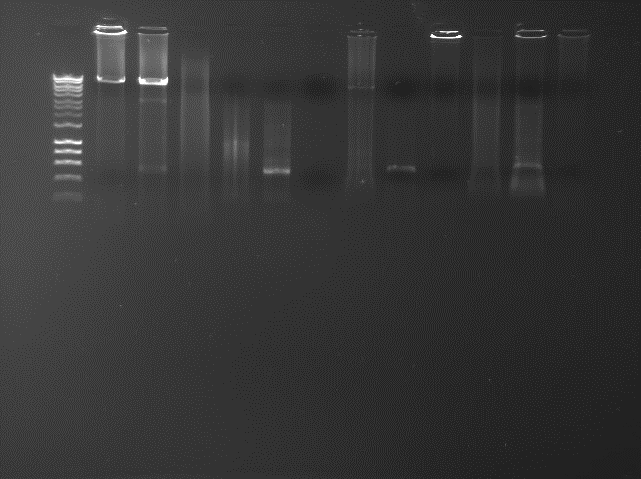


Takara

M 1 2 3


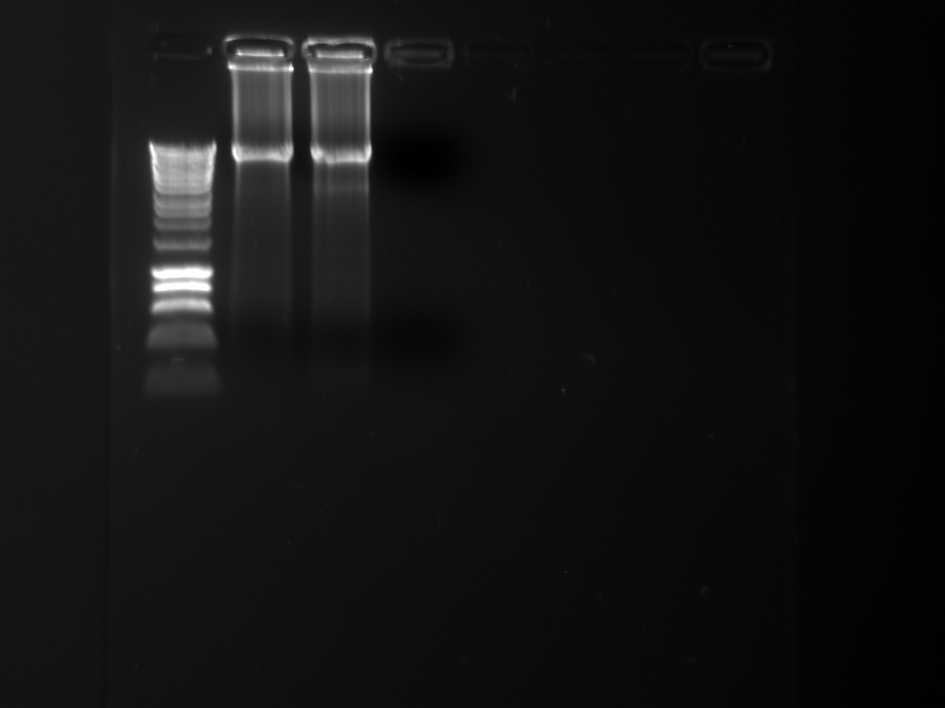


**Supplementary 1.B)** Gel electrophoresis of near full-length genome PCR products as presented in Figure 2 B. M, HyperLadder 1kb; 1-4, known EV positives from NSW Health Pathology East virology diagnostic lab; 5, CVB3 Nancy; 6, H_2_O control. Although KlenTaq produced near full-length bands from EV positive samples, in this case non-specific bands were produced which can result from enzyme degradation associated with extended storage.

Takara KlenTaq

M 1 2 3 4 5 6 1 2 3 4 5 6


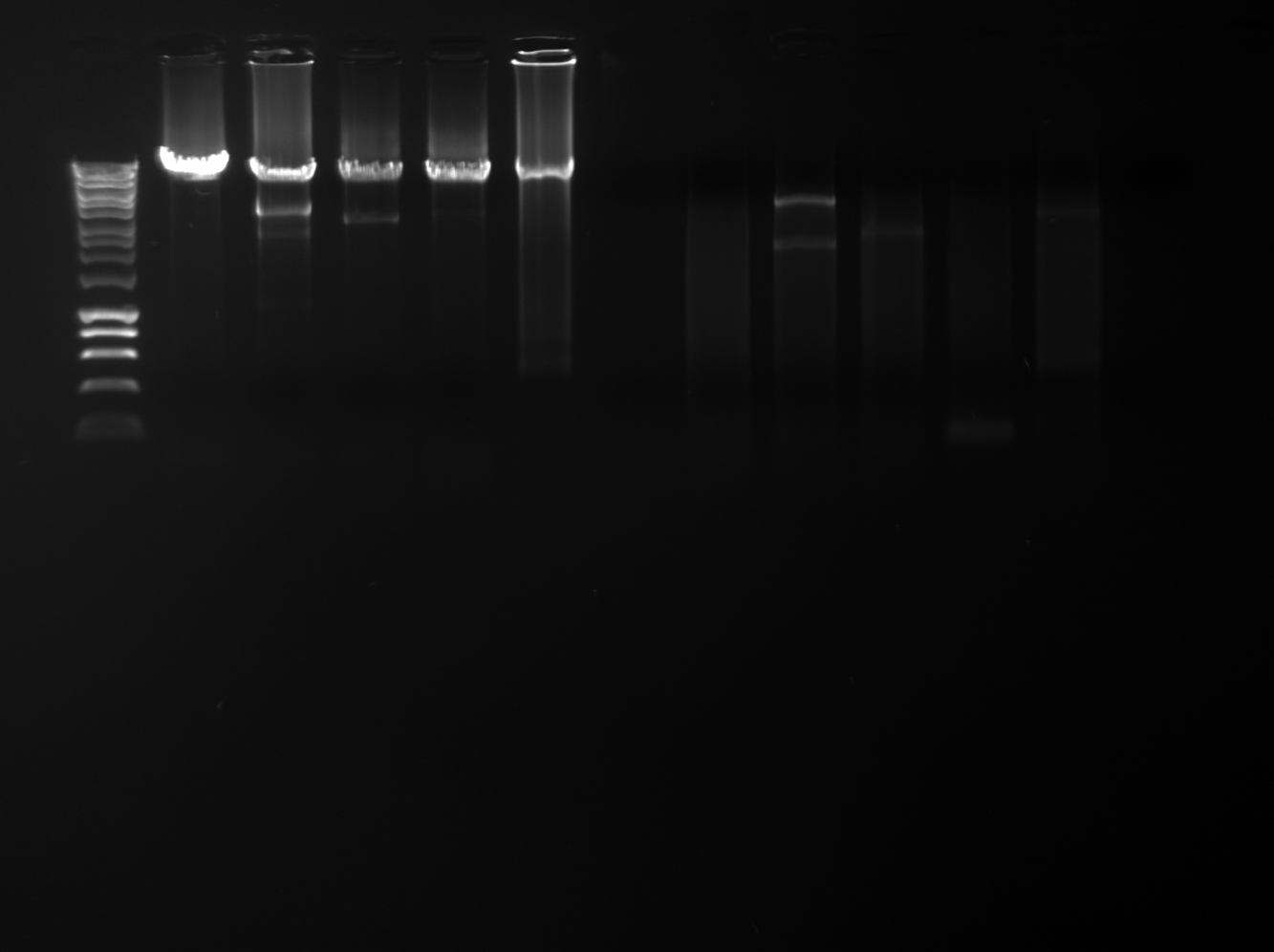


**Supplementary 2.** Protocol used for DNA polymerases during optimisation

For AccuTaq LA DNA Polymerase (Sigma) in both Round 1 and 2 PCR: Per reaction, 5 µL of refrigerated template cDNA/DNA, 5 µL of 10X AccuPrime PCR Buffer 1, 12 µL of 5 M Betaine, 1 µL of 10 mM dNTPs, 1 µL of 10 µM forward primer, 1 µL of 10 µM reverse primer, 24.5 µL of nuclease-free water and 0.5 µL of AccuPrime Taq HF Polymerase.

For KlenTaq LA (Clontech) in both Round 1 and 2 PCR: Per reaction, 5 µL of refrigerated template cDNA, 5 µL of 10X KlenTaq PCR Reaction Buffer, 12 µL of 5 M Betaine, 1 µL of 10 mM dNTPs, 1 µL of 10 µM forward primer, 1 µL of 10 µM reverse primer, 24 µL of nuclease-free water and 1 µL of 50X KlenTaq LA Polymerase Mix.

For PrimeSTAR GXL (Clontech) in both Round 1 and 2 PCR: Per reaction, 5 µL of refrigerated template cDNA/DNA, 10 µL of 5X PrimeSTAR GXL PCR Buffer, 12 µL of 5 M Betaine, 1 µL of 10 mM dNTPs, 1 µL of 10 µM forward primer, 1 µL of 10 µM reverse primer, 19 µL of nuclease-free water and 1 µL of PrimeSTAR GXL Polymerase.

For AccuTaq LA DNA Polymerase (Sigma), KlenTaq LA (Clontech) and PrimeSTAR GXL (Clontech):
Round 1 cycling conditions included 94°C for 2 min, 10 cycles at 94°C for 30 s, 55°C for 20 s, and 68°C for 8 min, 25 cycles at 94°C for 30 s, 57°C for 20 s, and 68°C for 8 min (+20 s per cycle), 68°C for 5 min. Round 2 cycling conditions included 94°C for 2 min, 10 cycles at 94°C for 30 s, 58°C for 20 s, and 68°C for 8 min, 25 cycles at 94°C for 30 s, 60°C for 20 s, and 68°C for 7 min 30 s (+20 s per cycle), 68°C for 5 min.

**Supplementary 3.** Genotyping results using Sanger sequencing of 5’UTR or VP1 region compared to near full-length genome next generation sequencing

| Sample | Genotype identified using Sanger | 5’UTR of VP1 region used | Genotype identified using near full-length genome sequencing | Agreement |
| --- | --- | --- | --- | --- |
| V05-2006-ECHO3 | ECHO3 | 5’UTR | ECHO3 | Yes |
| V06-2008-CVB2 | PV3 | 5’UTR | CVB2 | No |
| V07-2007-ECHO25 | ECHO11 | 5’UTR | ECHO25 | No |
| V08-2008-ECHO25 | CVB5 | 5’UTR | ECHO25 | No |
| V10-2008-CVA16 | CVA16 | VP1 | CVA16 | Yes |
| V13A-2008-ECHO18 | ECHO18 | 5’UTR | ECHO18 | Yes |
| V13B-2008-CVB3 | ECHO18 | 5’UTR | CVB3 | No |
| V14-2009-CVA9 | CVA9 | VP1 | CVA9 | Yes |
| V16-2005-CVB2 | EV71 | 5’UTR | CVB2 | No |
| V20-2008-CVA9 | PV3 | 5’UTR | CVA9 | No |
| V23-2007-CVA10 | CVA10 | 5’UTR | CVA10 | Yes |
| V26-2007-CVB5 | CVB5 | 5’UTR | CVB5 | Yes |
| V27-2006-CVA6 | CVA10 | 5’UTR | CVA6 | No |
| V28-2007-ECHO7 | ECHO18 | 5’UTR | ECHO7 | No |
| V33-2006-CVB2 | CVA22 | 5’UTR | CVB2 | No |
| V34-2008-CVA2 | CVA9 | 5’UTR | CVA2 | No |
| V35A-2006-ECHO7 | ECHO18 | 5’UTR | ECHO7 | No |
| V35B-2006-CVB3 | ECHO18 | 5’UTR | CVB3 | No |
| V38A-2009-CVB3 | CVA4 | 5’UTR | CVB3 | No |
| V38B-2009-ECHO7 | CVA4 | 5’UTR | ECHO7 | No |
| V39-2009-CVB3 | CVA4 | 5’UTR | CVB3 | No |
| V40-2006-CVB3 | EV-C | 5’UTR | CVB3 | No |
| V42-2008-CVB2 | CVB2 | VP1 | CVB2 | Yes |
| V43-2006-CVA16 | CVA16 | 5’UTR | CVA16 | Yes |
| V44-2008-CVA2 | CVA22 | 5’UTR | CVA2 | No |
| V45-2005-CVA2 | PV3 | 5’UTR | CVA2 | No |
| V46-2008-ECHO30 | ECHO30 | 5’UTR/VP1 | ECHO30 | Yes |
| V48-2008-CVA2 | CVA2 | VP1 | CVA2 | Yes |
| V50-2008-CVB5 | EV71 | 5’UTR | CVB5 | No |
| V51-2008-CVB5 | CVB5 | 5’UTR | CVB5 | Yes |
| V56-2006-CVA9 | PV3 | 5’UTR | CVA9 | No |

**Supplementary 4.** GenBank accession numbers for 70 sequenced enteroviruses

| Sample | Year of Collection | Genotype | Accession |
| --- | --- | --- | --- |
| NSW-V01-2005-PV3 | 2005 | PV3 | MF678293 |
| NSW-V03-2008-CVA19 | 2008 | CVA19 | MF678294 |
| NSW-V06-2008-CVB2 | 2008 | CVB2 | MF678295 |
| NSW-V07-2007-ECHO25 | 2007 | ECHO25 | MF678296 |
| NSW-V08-2008-ECHO25 | 2008 | ECHO25 | MF678297 |
| NSW-V09-2008-ECHO25 | 2008 | ECHO25 | MF678298 |
| NSW-V10-2008-CVA16 | 2008 | CVA16 | MF678299 |
| NSW-V11-2010-CVB4 | 2010 | CVB4 | MF678300 |
| NSW-V13A-2008-ECHO18 | 2008 | ECHO18 | MF678301 |
| NSW-V13B-2008-CVB3 | 2008 | CVB3 | MF678302 |
| NSW-V14-2009-CVA9 | 2009 | CVA9 | MF678303 |
| NSW-V15-2012-CVB3 | 2012 | CVB3 | MF678304 |
| NSW-V16-2005-CVB2 | 2005 | CVB2 | MF678305 |
| NSW-V17-2007-CVB2 | 2007 | ECHO6 | MF678306 |
| NSW-V18-2006-ECHO6 | 2006 | CVB2 | MF678307 |
| NSW-V19-2008-CVB5 | 2008 | CVB5 | MF678308 |
| NSW-V20-2008-CVA9 | 2008 | CVA9 | MF678309 |
| NSW-V21-2010-CVA2 | 2010 | CVA2 | MF678310 |
| NSW-V22-2009-CVB3 | 2009 | CVB3 | MF678311 |
| NSW-V23-2007-CVA10 | 2007 | CVA10 | MF678312 |
| NSW-V24-2008-CVA16 | 2008 | CVA16 | MF678313 |
| NSW-V25-2015-CVB3 | 2015 | CVB3 | MF678314 |
| NSW-V26-2007-CVB5 | 2007 | CVB5 | MF678315 |
| NSW-V28-2007-ECHO7 | 2007 | ECHO7 | MF678316 |
| NSW-V29-2008-CVB2 | 2008 | CVB2 | MF678317 |
| NSW-V30-2012-CVA6 | 2012 | CVA6 | MF678318 |
| NSW-V31-2010-CVB4 | 2010 | CVB4 | MF678319 |
| NSW-V32-2008-ECHO9 | 2008 | ECHO9 | MF678320 |
| NSW-V33-2006-CVB2 | 2006 | CVB2 | MF678321 |
| NSW-V34-2008-CVA2 | 2008 | CVA2 | MF678322 |
| NSW-V35A-2006-ECHO7 | 2006 | ECHOE7 | MF678323 |
| NSW-V35B-2006-CVB3 | 2006 | CVB3 | MF678324 |
| NSW-V36-2012-ECHO7 | 2012 | ECHO7 | MF678325 |
| NSW-V37-2013-CVB3 | 2013 | CVB3 | MF678326 |
| NSW-V38A-2009-CVB3 | 2009 | CVB3 | MF678327 |
| NSW-V39-2009-CVB3 | 2009 | CVB3 | MF678328 |
| NSW-V40-2006-CVB3 | 2006 | CVB3 | MF678329 |
| NSW-V41-2013-CVA9 | 2013 | CVA9 | MF678330 |
| NSW-V42-2008-CVB2 | 2008 | CVB2 | MF678331 |
| NSW-V43-2006-CVA16 | 2006 | CVA16 | MF678332 |
| NSW-V44-2008-CVA2 | 2008 | CVA2 | MF678333 |
| NSW-V45-2005-CVA2 | 2005 | CVA2 | MF678334 |
| NSW-V46-2008-ECHO30 | 2008 | ECHO30 | MF678335 |
| NSW-V47A-2005-ECHO9 | 2005 | ECHO9 | MF678336 |
| NSW-V47B-2005-ECHO30 | 2005 | ECHO30 | MF678337 |
| NSW-V48-2008-CVA2 | 2008 | CVA2 | MF678338 |
| NSW-V49-2012-ECHO7 | 2012 | ECHO7 | MF678339 |
| NSW-V50-2008-CVB5 | 2008 | CVB5 | MF678340 |
| NSW-V51-2008-CVB5 | 2008 | CVB5 | MF678341 |
| NSW-V52-2008-CVB2 | 2008 | CVB2 | MF678342 |
| NSW-V53-2010-CVB2 | 2010 | CVB2 | MF678343 |
| NSW-V54-2005-ECHO9 | 2005 | ECHO9 | MF678344 |
| NSW-V55-2005-PV2 | 2005 | PV2 | MF678345 |
| NSW-V56-2006-CVA9 | 2006 | CVA9 | MF678346 |
| NSW-V57-2007-CVB4 | 2007 | CVB4 | MF678347 |
| NSW-V58-2010-ECHO25 | 2010 | ECHO25 | MF678348 |
| NSW-V02-2011-ECHO18 | 2011 | ECHO18 | MF838733 |
| NSW-V04-2012-ECHO14 | 2012 | ECHO14 | MF838734 |
| NSW-V05-2006-ECHO3 | 2006 | ECHO3 | MF838735 |
| NSW-V27-2006-CVA6 | 2006 | CVA6 | MF838736 |
| NSW-V38B-2009-ECHO7 | 2009 | ECHO7 | MF838737 |
| NSW-V12-2008-CVB5 | 2008 | CVB5 | MF962897 |
